# Early reading at first grade predicts adult reading at age 42 in typical and dyslexic readers

**DOI:** 10.1038/s41539-023-00205-7

**Published:** 2023-11-28

**Authors:** Emilio Ferrer, Bennett A. Shaywitz, John M. Holahan, Sally E. Shaywitz

**Affiliations:** 1 Department of Psychology, University of California, Davis, CA USA; 2 Yale Center for Dyslexia & Creativity, Yale University School of Medicine, Yale, CT USA

**Keywords:** Human behaviour, Education

## Abstract

Research indicates that the achievement gap in reading between typical and dyslexic readers is already evident in first grade and persists through adolescence. However, it is not known whether this reading gap persists into adult life. In this report we use an epidemiologic sample of 312 children (typical readers = 246; dyslexic readers = 66), followed longitudinally from age 5 through adulthood and examine two fundamental questions: 1) Is reading level in 1^st^ grade predictive of reading proficiency in adulthood in typical and dyslexic readers? and 2) Are the *trajectories* of reading development from 1^st^ through 5^th^ grade predictive of reading proficiency in adulthood in typical and dyslexic readers? Our findings indicate that early reading levels in 1^st^ grade as well as the trajectory of reading development through the first five years of school were associated with reading scores in adulthood. This association was stronger for dyslexic than for typical readers, especially the latter factor. These findings indicate that the achievement gap between typical and dyslexic readers persists far beyond adolescence, in fact, into adult life.

## Introduction

Strong evidence indicates that the achievement gap in reading between typical and dyslexic readers occurs as early as first grade and persists through adolescence^1^, a finding consonant with the Cunningham and Stanovich report that reading as early as 1^st^ grade is a strong predictor of reading-related tasks (reading comprehension, vocabulary, and general knowledge) 10 years later, even after controlling for measures of cognitive ability^2^. However, the future course of the achievement gap is not known, for example, its persistence and trajectory into adult life.

In the orthographically regular Finnish, approximately only one in five dyslexic individuals succeed in compensating for their underlying difficulties and develop adequate reading skills by adulthood^3^. However, little is known about the trajectory of reading development into adulthood in English speakers and readers. In particular, we do not know if the reading trajectory during the early grades helps to predict reading proficiency later in life, especially for dyslexic readers, who all too frequently continue to struggle with reading as adults. Such information will be critical in planning effective and meaningful interventions and accommodations for dyslexic readers not only during the school years but into adult life as well.

In this report we examine these questions using a unique population, an epidemiologic sample of 312 children, including typical readers (*n* = 246) and dyslexic readers (*n* = 66), followed longitudinally from age 5 through adulthood. Our study addresses two fundamental questions: 1) Is reading level in 1^st^ grade predictive of reading proficiency in adulthood in typical and dyslexic readers? and 2) Are the *trajectories* of reading development from 1^st^ through 5^th^ grade predictive of reading proficiency in adulthood in typical and dyslexic readers?

Table 1 reports results from analyses involving growth curve models^4–6^ capturing changes in reading from 1^st^ to 5^th^ grade together with predictions from such changes and early levels onto reading measures taken during adulthood (average age 42 years). The first part of Table 1 includes the features of the growth curve model for both typical and dyslexic readers. The mean intercept represents the average reading level at 1^st^ grade, indicating large differences between both groups (*µ*_0_ = 13.62 and 6.92, for typical and dyslexic readers, respectively). The mean slope denotes the mean overall increase in reading scores from 1^st^ through 5^th^ grade (*µ*_s_ = 12.00 and 13.10, for typical and dyslexic readers, respectively). Given the large discrepancy in reading levels at 1^st^ grade between typical and dyslexic readers together with their similar increases over the five grades, the reading levels at 5^th^ grade remain disproportionate between both groups.

**Table 1:** Parameter estimates predicting ART-2 from WJ reading in grades 1–5.

	Typical estimate (SE) *p*	Dyslexic estimate (SE) *p*
*WJ Reading*		
Mean intercept *µ*_0_	13.62 (0.288) 0.000	6.92 (0.427) 0.000
Mean slope *µ*_s_	12.00 (0.231) 0.000	13.10 (0.493) 0.000
Variance intercept *σ*^2^_0_	18.65 (1.81) 0.000	9.69 (0.427) 0.000
Variance slope *σ*^2^_s_	9.67 (1.14) 0.000	13.10 (0.493) 0.000
Correlation *ρ*_0s_	−0.769 (0.031) 0.000	−0.418 (0.122) 0.000
Residual variance *σ*^2^_e_	1.77 (0.092) 0.000	2.34 (0.235) 0.000
*Predictions of ART-2*		
*ATOTACC*		
*β*_0_ →	0.980 (0.072) 0.000	0.473 (0.112) 0.000
*β*_s_ →	0.741 (0.086) 0.000	0.832 (0.089) 0.000
*R*^*2*^	0.511	0.610
*ATOTCOMP*		
*β*_0_ →	0.545 (0.096) 0.009	0.545 (0.119) 0.000
*β*_s_ →	0.280 (0.107) 0.009	0.567 (0.116) 0.000
*R*^*2*^	0.101	0.361
*AAVGSPEED*		
*β*_0_ →	0.653 (0.091) 0.000	0.460 (0.127) 0.000
*β*_s_ →	0.361 (0.103) 0.000	0.520 (0.118) 0.000
*R*^*2*^	0.194	0.320
*STOTCOMP*		
*β*_0_ →	0.554 (0.094) 0.000	0.561 (0.120) 0.000
*β*_s_ →	0.199 (0.105) 0.058	0.519 (0.119) 0.000
*R*^*2*^	0.180	0.353
*SAVGSECS*		
*β*_0_ →	−0.376 (0.100) 0.000	−0.400 (0.134) 0.003
*β*_s_ →	−0.078 (0.110) 0.480	−0.355 (0.136) 0.009
*R*^*2*^	0.103	0.168

Note. *N Typical* = 246, *N Dyslexic* = 66. Maximum Likelihood estimates. *SE* standard errors. Mean intercept (*µ*_0_) = average value of *WJ Reading* at 1^st^ grade; Mean slope (*µ*_s_) = average change in *WJ Reading* from 1^st^ to 5^th^ grade. *WJ* Woodcock–Johnson psycho-educational battery, *ATOTACC* total accuracy raw score, *ATOTCOMP* total comprehension raw score, *AAVGSPEED* words per minute for passages, *STOTCOMP* total comprehension raw score (silent reading), *SAVGSPEED* average seconds to read passages raw score (silent reading).

The variances of the intercept and slope (*σ*^2^_0_ and *σ*^2^_s_) represent variation across individuals in the level and slope. Whereas typical readers show more variation in reading levels in 1^st^ grade than dyslexic do (*σ*^2^_0_ = 18.65 vs. 9.69), dyslexic readers show more variation in reading development from 1^st^ to 5^th^ grade (*σ*^2^_s_ = 9.67 and 13.10).

The second part of Table 1 includes results from the regression analyses predicting reading outcomes in adulthood as measured using an adult test of reading (ART-2) from the intercept and slope of the reading growth curves from 1^st^ to 5^th^ grade. For example, for Total Accuracy, as measured by Raw Score, (*ATOTACC)*), the intercept from the growth curve model (reading level at 1^st^ grade) was predictive of accuracy scores in adulthood for both typical and dyslexic groups, although more strongly for typical readers (*β*_0_ = 0.980 and 0.473, for typical and dyslexic readers, respectively). Similarly, the slope from the growth curve model (changes in reading from 1^st^ to 5^th^ grade) was also predictive of accuracy scores in adulthood for both groups, yet slightly more strongly for dyslexic readers (*β*_s_ = 0.741 and 0.832, for typical and dyslexic readers, respectively). Finally, the third entry indicates the amount of variance in the adult outcome measure explained by the intercept and slope. For *ATOTACC*, these values are high for both groups (*R*^2^ = 0.0511 and 0.610, for typical and dyslexic readers, respectively).

**Fig. 1:**
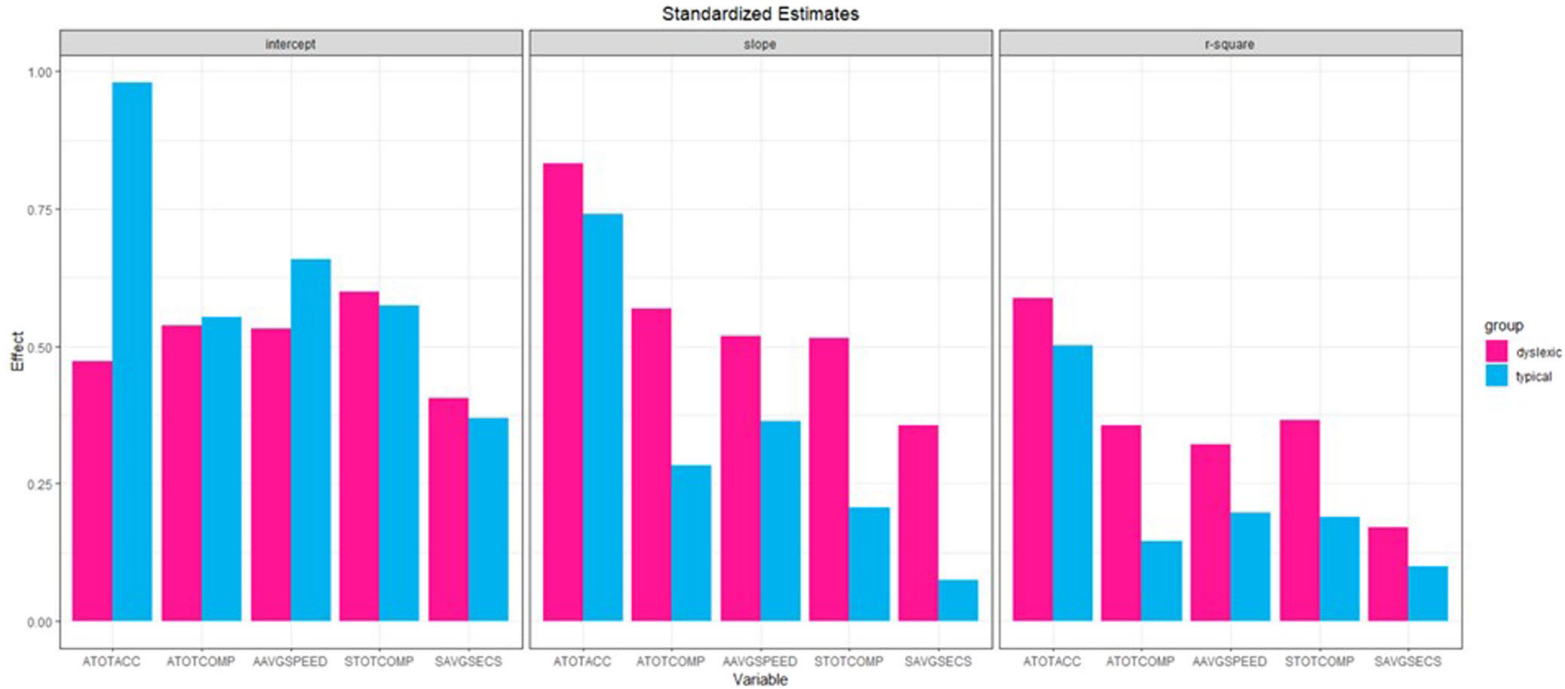
Standardized regression estimates predicting adult literacy measures and corresponding *R*-squared values. **A (Left).** Standardized regression estimates predicting adult literacy measures from reading scores in 1^st^ grade. Pink bars represent coefficients from Dyslexic readers; blue bars represent coefficient from Typical readers. **B (Middle).** Standardized regression estimates predicting adult literacy measures from slope coefficients representing change in reading scores from 1^st^ to 5^th^ grade. Pink bars represent coefficients from Dyslexic readers; blue bars represent coefficient from Typical readers. **C (Right).** *R*-square values for the adult literacy measures from regression models. ATOTACC total accuracy raw score, ATOTCOMP total comprehension raw score, AAVGSPEED words per minute for passages, STOTCOMP total comprehension raw score (silent reading), SAVGSPEED average seconds to read passages raw score (silent reading).

For the rest of the reading outcomes in adulthood, the predictions followed a consistent pattern. The relations from the intercept were strong and relatively similar between typical and dyslexic readers. The relations from the slope, however, were weaker (or nonsignificant) for typical readers but remained strong for dyslexic readers. For example, for Total Comprehension, as measured by Raw Score (*ATOTCOMP)*, the relation from reading levels at 1^st^ grade were equivalent for both groups (*β*_0_ = 0.545). In contrast, whereas the relation from the slope decreased for typical readers (*β*_s_ = 0.280), it increased for dyslexic readers (*β*_s_ = 0.567). In other words, reading level in 1^st^ grade was associated with comprehension scores in adulthood similarly for both groups. However, the trajectory of children’s reading development from 1^st^ to 5^th^ grade was strongly related to adult comprehension scores for dyslexic, but not as much for typical readers. Although this pattern was true for all remaining outcomes, it was even more pronounced for silent reading comprehension, as measured by raw score (*STOTCOMP*), and Average Seconds to Read Passages during silent reading, as measured by Raw Score (*SAVGSECS)*, for which the prediction from the slope was not statistically significant for typical readers (*β*_s_ = 0.199 and −0.078, *p* > 0.05), but remained high for dyslexic readers (*β*_s_ = 0.519 and –0.355, *p* < 0.05). Contrary to the accuracy measure (*ATOTACC*), the *R*^2^ values for the remaining adult reading outcomes were substantially lower for typical readers and remained at moderate values for dyslexic readers. Figure 1 provides a visual summary of these results displaying the similar predictions from the intercept for both groups together with the stronger predictions from the slope as well as higher *R*^2^ values for dyslexic readers.

**Fig. 2:**
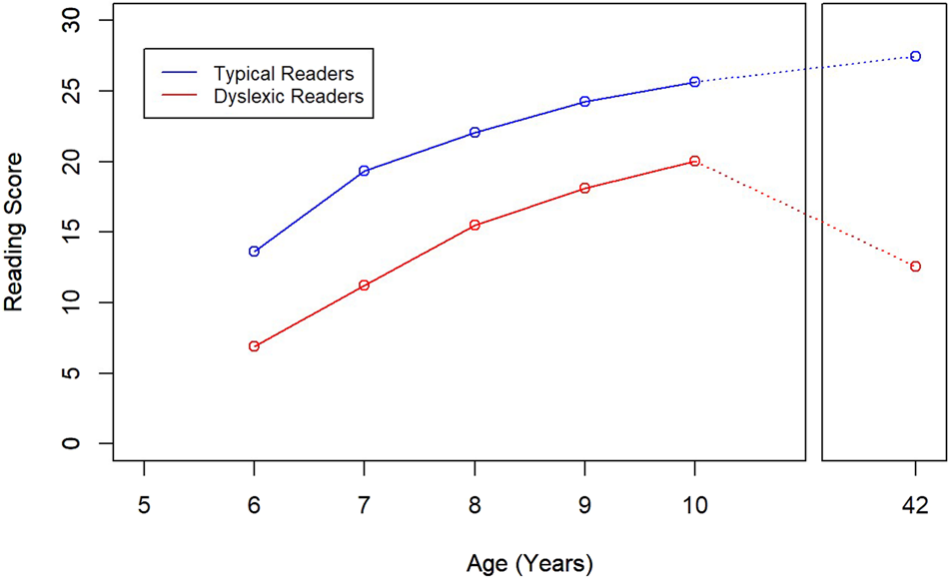
Reading trajectories from 1^st^ to 5^th^ grade (ages 6 to 10), and into adulthood (age 42) for Typical (blue) and Dyslexic (red) readers. The trajectories for years 6-10 correspond to the total reading composite scores from the *Woodcock-Johnson psycho-educational battery*. Values at age 42 represent *ATOTACC* total accuracy raw scores.

Together, our findings indicate that early reading levels in 1^st^ grade along with the trajectory of reading development through the first five years of school were associated with reading scores in adulthood. However, this association was stronger for dyslexic than for typical readers, especially the trajectory of changes in reading from 1^st^ to 5^th^ grade. Similarly, early reading levels and the trajectory of changes explained more of the variation observed in adult scores for dyslexic than for typical readers. These findings indicate that the achievement gap between typical and dyslexic readers persists far beyond adolescence, in fact, into adult life. They further suggest that, for dyslexic readers, reading in first grade and, in particular, the trajectory of reading during the first five school grades, is strongly related to reading in adulthood. This is illustrated in Fig. 2. The reading gap between typical and dyslexic readers that is already evident in 1^st^ grade never closes during the school years. When reading is examined in adulthood, the gap still remains. This study complements other similar studies following poor readers from adolescence into adulthood^4^. This important finding mandates early identification and early intervention to minimize and perhaps reverse the adult consequences of dyslexia while children are still in school.

Finally, our findings depart from assumptions by standardized testing agencies that require applicants requesting accommodations (i.e., extra time for dyslexic students) to be retested every three or five years. Our data indicate that once a person is identified as dyslexic in the early grades, he or she is very likely to be dyslexic as an adult at age 42. Thus, the requirement for retesting of dyslexic students who are adults every few years is not supported by our findings.

## Methods

### Participants

We report findings from The Connecticut Longitudinal Study^5^, an epidemiologic sample survey of schoolchildren representative of children entering public kindergarten. Of the 312 participants with complete data, 52.8% are females and 47.2% males. The sample contains Caucasians (85.2%), African Americans (11.8%), Asians (1.0%), Hispanics (2.0%), and other children with unknown ethnicity (0.3%). The composition of this sample was similar to the racial and ethnic composition of the U.S. at the time of the study. All participants were primary English speakers. This cohort, assembled from a 2-stage probability sample, has been followed longitudinally from school entry into adulthood to study the development of reading. The study was approved by the Yale University Human Investigation Committee and IRB, and informed consent was obtained from parents of all participants.

### Measures

Reading skills during grade school were measured using the WJ Reading Cluster (composite of Letter-Word Identification, Word Attack, and Passage Comprehension subtests) from the Woodcock-Johnson Psycho-Educational Test Battery^6^. Reading during adulthood was measured using the ART-2 Adult Reading Test^7^. The ART-2 measures prose reading accuracy, reading comprehension (silent and aloud) and speed of reading (silent and aloud). Table 2 reports descriptive statistics of reading measures from WJ-R and ART-2, for both typical and dyslexic readers. Across all measures, both grade school and adult reading, typical readers have higher means than dyslexic readers (Note that *SAVGSECS* is time needed to read a passage. Thus, higher values indicate slower reading).

**Table 2:** Descriptive Statistics.

	Typical mean (SD)	Dyslexic mean (SD)
*WJ Reading*		
Grade 1	13.64 (4.47)	6.84 (3.28)
Grade 2	19.33 (3.93)	11.32 (3.21)
Grade 3	22.03 (3.25)	15.51 (3.77)
Grade 4	24.29 (3.02)	18.06 (3.60)
Grade 5	25.62 (2.87)	20.03 (3.70)
*ART-2*		
ATOTACC	27.48 (10.3)	12.58 (9.15)
ATOTCOMP	12.70 (4.49)	9.76 (5.55)
AAVGSPEED	144.36 (19.7)	120.7 (26.2)
STOTCOMP	14.40 (4.18)	11.65 (4.53)
SAVGSECS	96.21 (29.4)	121.5 (35.3)

Note. *N Typical* = 246, *N Dyslexic* = 66. *WJ* *Woodcock-Johnson psycho-educational battery*, *ATOTACC* total accuracy raw score, *ATOTCOMP* total comprehension raw score, *AAVGSPEED* words per minute for passages, *STOTCOMP* total comprehension raw score (silent reading), *SAVGSPEED* average seconds to read passages raw score (silent reading).

### Criteria for dyslexia

Dyslexia was defined using the WJ Reading Cluster scores and the WISC-R Full Scale IQ score^8^. Dyslexia was determined if a participant met criteria based on either low achievement (reading cluster age standard score < 90) or IQ-achievement discrepancy criteria (a reading cluster > 1.5 standard deviations lower than that predicted by Full Scale IQ) in grades 2 or 4^2^. Both definitions validly identify children as poor readers, and there is little evidence of differences between subgroups formed by one definition versus the other^3^. This definition of dyslexia status yielded a Dyslexic Readers Group and a Typical Readers group.

### Statistical analysis

To examine changes in reading from 1^st^ to 5^th^ grade, we used a growth curve model^9–11^. This model allowed us to characterize the changes in reading scores across the five grades in terms of an intercept and a slope, together with variance components in both parameters, representing variation across individuals in the intercept and the slope.

A basic growth curve model for a variable *Y* measured over time or grade (*t* = 1 to *T*) on the same individual (*n* = 1 to *N*) can be written as

1 $${Y}_{{tn}}={y}_{0n}+{B}_{t}\,{{\cdot }}\,{y}_{{sn}}+{e}_{{tn}},$$

where *y*_*0*_ is a latent score representing an individual’s initial level, *B*_*t*_ is the group “basis” coefficients that represent the timing or shape of the growth, *y*_*s*_ is a latent score representing the slope, or the individual change over time, and *e*_*t*_ represents the unobserved error of measurement. As basis coefficients, we used *B*_*t*_ = [0, *B*_2_, *B*_3_, *B*_4_, 1]. These coefficients allowed us to identify the intercept as the reading scores at 1^st^ grade and the slope as the overall changes in reading from 1^st^ to 5^th^ grade. The remaining coefficients *B*_2-4_ were estimated from the data, each representing the percentage of the changes from the overall slope. This specification allowed us to capture possible nonlinearities in the changes across grades. In addition, this model includes sources of individual differences in the level and slope, whose terms can be decomposed at a second level as

2 $$\begin{array}{l}{y}_{0n}={{\mu }}_{0}+{e}_{0{\rm{n}}},{\rm{and}}\\{y}_{{sn}}={{\mu }}_{{\rm{s}}}+{e}_{{\rm{sn}}},\end{array}$$

where the level and slope scores have fixed group means (*μ*_0_ and *μ*_s_) and residuals (*e*_*0n*_ and *e*_sn_), and these residuals have variance components (*σ*_*0*_^*2*^, *σ*_*s*_^*2*^ and *σ*_*0s*_) but are assumed to have zero means and to be normally distributed.

To predict the adult reading outcomes, we expanded this model by including regression coefficients from the intercept and slope of the growth model directly onto each of the outcomes (separately, for each outcome). Finally, we examined differences between typical and dyslexic readers using a multiple-group model^11,12^. This procedure allowed us to estimate differences between the groups in both the parameters of the growth model and their predictions of the adult reading outcomes.

### Reporting summary

Further information on research design is available in the Nature Research Reporting Summary linked to this article.

### Supplementary information

Reporting Summary

## Data Availability

The datasets generated during and/or analyzed during the current study are not publicly available due to containing information that could compromise research participant privacy/consent but are available from the corresponding author on reasonable request indicating the goals of the intended use.
