# Author Correction: Early reading at first grade predicts adult reading at age 42 in typical and dyslexic readers

**DOI:** 10.1038/s41539-023-00212-8

**Published:** 2024-01-10

**Authors:** Emilio Ferrer, Bennett A. Shaywitz, John M. Holahan, Sally E. Shaywitz

**Affiliations:** 1 Department of Psychology, University of California, Davis, CA USA; 2 Yale Center for Dyslexia & Creativity, Yale University School of Medicine, Yale, CT USA

**Keywords:** Human behaviour, Education

Correction to: *npj Science of Learning* 10.1038/s41539-023-00205-7, published online 28 November 2023

The Acknowledgements section was incomplete from this article and should have read We thank the members of the DIPS lab at UC Davis for their input and Samuel Aragones for his help with the graphics.

